# The Statistics of Justice

**Published:** 1859-01-01

**Authors:** 


### Art. V.—THE STATISTICS OF JUSTICE.

When, in March, 1856, Lord Brougham addressed the House of Lords upon the "great subject" (as he phrased it) of Judicial Statistics, he explained the meaning of that term in the following words:—"It signifies the regular and constant record of the whole particulars connected with the administration of the law in all its branches; its administration by all courts civil and criminal, general and local; the state of those courts as to judges and other office bearers; their whole proceedings through every stage; together with every matter concerning the working of the law, though not having come within the cognizance of any tribunal—in a word, the record, in minute detail, and for the most part in a tabular form, of all the facts connected with the execution of our laws. Need there be more said to show," continued his Lordship, "I will not say the great value, but the permanent importance—nay, the absolute necessity, of this knowledge to the makers of these laws? Can we honestly and safely exercise our legislative functions without them—can we test the influence of our law-making without them—whether we should persist or not in the steps we have taken? Jurisprudence is eminently a practical science. It must be carried on with constant reference to the effects it has produced . . . . Full and minute statistical details are to the lawgiver, as the chart, the compass, and the lead to the navigator." *

There is, however, another application of judicial statistics, and one which, in some respects, is scarcely less important than that which refers to legislation. They furnish concerning several of the most momentous questions in moral and social science the only reliable data, without which data we cannot hope to arrive at any satisfactory knowledge respecting the distribution, the increase and the decrease of crime; the causes which lead to its variations in amount in different localities and at different times; the influence of education, of punishment, and of reformatory measures in its repression; the prevalence, fluctuations, and criminal affinities of drunkenness and of prostitution; the extent of juvenile crime and of vagrancy; the degree in which our commercial dealings are infected with fraudulent and illegal transactions; and the intensity with which different classes of the population are affected by criminal influences. Judicial statistics, in short, must form the surest foundation of all inquiry into the moral and social condition of the whole or of any portion of a nation; because no clearer index, or one less liable to misappre-

* Hansard, vol. cxl. p. 1674.

hension, can exist of the moral state of a well-governed nation than the records of its courts of civil and criminal judicature.

Moreover, we may anticipate that these records will, in addition to the elucidation of the concrete moral and social questions referred to, furnish data by which the more purely psychical phenomena involved in them may, in several of their most interesting aspects, be investigated. Those obscure, yet gravely important epidemic manias of murder, of suicide, of infanticide, of fraudulent commercial transactions, and of other criminal offences, which appear to prevail from time to time, apart from what may be called the ordinary fluctuations of criminal acts, can, in the first instance, be examined only by the aid of systematic and permanent statistical records. Such information as we now possess upon any one of these so-called epidemic manias, has been derived, perhaps with one or two exceptions, from vague observations made at the time of the supposed prevalence; the state and condition of the specific form of crime in the interval being almost altogether unknown or neglected. It may be assumed that any extraordinary phenomenon occurring in the progress of a series of events cannot be successfully, or even correctly investigated, until the ordinary phenomena of the series have been determined; hence the necessity which exists for the careful registration upon a systematic and well-digested plan of all the ascertainable facts of a series. This statistical method must underlie all severe modes of investigation, in whatever manner these may subsequently be modified, linked together, and developed by hypothesis arising out of the conclusions to which the first portions of any series of observations or facts may appear to point.

Hitherto the judicial statistics which we have possessed in England, unlike those of France, Belgium, and other continental states, have been very fragmentary, incomplete, and difficult of access. The importance (if, indeed, the importance be yet fully felt) of a systematic registration of the proceedings of justice has not been admitted either by the Government or by the people of this country until very lately. For the statistics which we possessed previous to the year 1856, we were dependent mainly upon the voluntary efforts of a few individuals, and upon the spasmodic requests of members of Parliament for data, as the necessity might arise during the sittings of Parliament. In 1857, however, a Blue Book was issued by Government headed "Judicial Statistics," but containing only the statistics of criminal justice in England and Wales for the previous year; and last year another Blue Book was issued under the same heading, but which, in addition to the statistics of criminal justice for 1857, contained the outlines of a scheme for the collection of the statistics of the Common Law,

Equity, Civil and Canon Law Courts. The history of these Blue Books is somewhat curious and interesting, and we add a brief account of it, as a matter of justice to the gentleman to whom we are most indebted for them.

When the late Mr. Porter was appointed to the head of the Statistical Department of the Board of Trade, he represented to the Secretary of State the want that then existed of any effective statistics of crime and punishment. Mr. Samuel Redgrave, an accomplished statist, at that time and since attached to the Home Office, by the Secretary of State's directions, undertook to remedy the defect, and early in 1835 a series of criminal tables for the preceding year were published upon a classification and arrangement prepared and carried out by Mr. Redgrave. This arrangement and form of issue was continued, in nearly the same form, year by year, until 1855; but in the meantime a gradually increasing interest had been manifested on all hands respecting the criminal and vagrant classes of the population, prison discipline, and other questions connected with punishment and crime, and it had become manifest that the criminal tables published by Government, at first little heeded, but now of admitted value, were not sufficiently comprehensive. This arose from no fault of the compiler, but partly from the parsimony of the Government in objecting to the insertion of more than the barest details, on account of expense in printing (although the only expense to which the Government was placed for these tables was the printing), and partly from the imperfect character of the returns from which they had to be compiled. Towards the termination of 1855, Mr. Redgrave submitted to the Government a plan in detail for the enlargement of the Home Office Statistics of Justice; and early in 1856, Lord Ebrington, at the request of the English gentlemen who attended the Statistical Congress in Paris the preceding autumn, addressed a letter to Lord Palmerston, on the deficiency of the statistical organization in the different departments of the State; and this letter was ordered to be printed by the House of Lords, together with a letter addressed by Professor Leone Levi to A. W. Fonblanque, Esq., of the Statistical Department of the Board of Trade, on the Judicial Statistics of the United Kingdom as compared with the French Civil, Commercial, and Criminal Statistics. In March, 1856, Lord Brougham submitted to the House of Lords a Bill to provide for the collection of the statistics of justice, and he made a very eloquent and powerful appeal to the House upon the necessity of the measure. Lord Brougham's Bill was not proceeded with, but the Government was moved by the different proceedings mentioned, and Sir George Grey, then Secretary of State for the Home Department, directed Mr. Redgrave to carry out the plan which he had submitted to the Government in the previous year, and the result of that gentleman's labours, up to the present time, are to be found in the Blue Books to which we have already referred.

It is difficult to estimate rightly the labour and thought which have been involved in carrying out, even in its present incomplete state, Mr. Redgrave's great scheme. Some notion of his work may be formed when we state that he has not had simply to arrange and place in comprehensive connexion statistics which had previously existed, or the means for obtaining which were ready to hand, but in the majority of instances he has had to make the necessary arrangements for the collection of the details the results of which he has laid, or is about to lay, before us. Some little time longer must elapse before his invaluable work can be carried out to the extent which he proposes; and it is worthy of being remarked, that for many of the returns from which his statistics are derived, he has to depend solely upon the good feeling of the officers of justice who furnish them, and that his own labour is simply one of love, undertaken, we believe, in addition to his more specific duties.

Whether it is wise to allow work of such incalculable importance to the community to depend in any degree upon voluntary contributions, or that the founder and chief worker should receive only such honour (flattering as it may be) as will be awarded to him by scientific circles, is somewhat more than doubtful.

The Blue Book of Judicial Statistics for 1857 (to which we shall confine our attention) is divided into two parts. The first contains, (1) the returns of the Police and Constabulary; (2) the returns of Criminal Proceedings; and (3) the returns concerning Prisons, Reformatory Institutions, and Criminal Lunatics. The police returns are entirely new, and appear for the first time in the statistics for 1857; but these returns do not yet include the whole kingdom—a regular police force having still to be organized in several districts. The criminal proceeding returns are similar to those previously published under the title of Criminal Tables; and the prison returns present, for the first time, at one view, information previously scattered in several reports. The second part of this Blue Book contains copies of the forms which it is proposed to make use of in recording the statistics of the Common Law, Equity, Civil, and Canon Law Courts, when the arrangements for the collection of those statistics shall have been completed.

The following is a summary of the facts contained in this Blue Book which come within the scope of this Journal:—

In 1857, there were committed in England and Wales 57,273

crimes; 32,031 persons were apprehended; and 17,861 persons were committed or bailed for trial. Several persons often take part in a single crime, or several crimes may be committed by a single person. These, and a few other liabilities to error, occur in the returns, without the means for correcting them; but subject to these drawbacks, it would appear from the returns that great differences occur between the number of offences and the number of arrests in different classes of offences. In crimes against the person, the number of persons apprehended equals, and often exceeds, the number of offences committed; in attempts upon the dwelling, burglary, housebreaking, &c., the apprehensions were 2084, the number of offences committed, 5428; and in robbery and attempts to rob, 854 apprehensions occur, to 1029 offences committed.

The number of persons proceeded against summarily was 369,233, of whom 291,030 were males, 78,203 females. Of these individuals, 192,235 males, and 41,524 females, were convicted by the justices. The offences with which the large number of persons summarily dealt with were charged, "represent," Mr. Redgrave remarks, "the vices, rather than the crimes, of the population." The offence first in magnitude is assault, for which 36,300 males were convicted, and 8560 females. The arrests for this offence amounted to 76,029, of which 2584 were made for aggravated assaults on women and children; 12,750 for assaults on peace officers; and 60,695 for common assaults. Next in order of amount to assault stand drunkenness, and being drunk and disorderly. For these offences 44,894 persons were convicted, of whom 35,867 were males, and 9027 females. Following in order of amount, come petty thefts, for which 20,577 persons were convicted, 15,832 males and 4745 females; vagrancy, for which 18,023 persons were convicted, 10,302 males and 7721 females; offences punishable under Police Acts, for which 17,415 persons were convicted, 13,206 males and 4209 females; offences under Local Acts, and Bye-Laws, for which 16,703 persons were convicted, 15,533 males and 1170 females; offences under the Ways Acts, including the Turnpike, Highways, and Stage Carriage Acts, for which 14,226 persons were convicted, 14,044 males and 182 females; offences of wilful damage and trespass, for which 10,168 persons were convicted, 8341 males, and 1827 females; offences under the Licensed Victuallers' and Beer Acts, for which 9224 persons were convicted, 8376 males and 848 females; offences relating to servants, apprentices, or masters, for which 5373 persons were convicted, 5102 males and 271 females; and nuisances and offences against the health, for which 3107 persons were convicted, 2702 males and 405 females.

The character of the persons who were in the custody of the police during the year is shown in the following table:—

| Characters. | Males. | Females. | Total. |
|---|---|---|---|
| Known Thieves. . . . . . . . | 18,556 | 4,546 | 23,102 |
| Prostitutes . . . . . . . . . | — | 24,282 | 24,282 |
| Vagrants and Tramps . . . . | 14,272 | 4,998 | 19,270 |
| Suspicious Characters. . . . . | 40,112 | 6,692 | 46,804 |
| No known occupations . . . . | 5,218 | 1,696 | 6,914 |
| Previous good Characters . . . | 112,017 | 14,548 | 126,565 |
| Characters unknown and not ascertained . . . . . . . . . . | 124,257 | 30,070 | 154,327 |
| Total . . . | 314,432 | 86,832 | 401,264 |

"Upon the above large data," writes Mr. Redgrave, "it is shown that of those proceeded against by indictment 54·0 per cent. were of the criminal class, 19·1 per cent. of previous good character, and of 26·9 per cent. the characters were either unknown or were not ascertained. Of those proceeded against for the lesser offences determined by Justices 27·9 were of the criminal class, 32·6 per cent. were of previous good character, and of 39·5 per cent. the characters were unknown or unascertained. Altogether 120,372 persons of the criminal class or suspected to belong to it were in the hands of the police in the year 1857, and of these 24,282 were prostitutes. What proportion these large numbers bear to the whole class which they represent, there are at this time little means of determining. In the last Census 304,109 persons were described as in criminal occupations, that is, as vagrants or persons of no stated employments, but these are probably very much under the mark. It would be useless, however, to enlarge upon this question, though one of great interest, as I hope next year, by the aid of the police, to include in these statistics a census of the criminal class at large, including all known thieves and depredators, receivers of stolen goods, prostitutes, suspected persons, and vagrants and tramps, and to show the number in the jurisdiction of each separate Police Force, with the number and class of their houses of resort."—(p. x.)

The coroners' returns (which are included in the police returns) show that 20,157 inquests were held in 1857; 13,941 upon males, and 6216 upon females. These numbers show a decrease of 2064 inquests, upon the previous year. The cause of this decrease is not very apparent, unless it is to be attributed to the more close control exercised by the Quarter Sessions, in the disallowance of the costs of inquests, which the court deem to have been unnecessarily held. The findings of the juries were as follows:—

| | Total. | Proportion per cent. |
|---|---|---|
| Murder | 184 | ·91 |
| Manslaughter | 187 | ·93 |
| Justifiable homicide | 6 | ·03 |
| Suicide, or self-murder | 1349 | 6·69 |
| Accidental death | 8930 | 44·30 |
| Injuries, causes unknown | 237 | 1·18 |
| Found dead | 2949 | 14·63 |
| Natural death:— | | |
| From excessive drinking | 323 | 1·60 |
| Disease aggravated by neglect | 143 | ·71 |
| Want, cold, exposure, &c. | 167 | ·83 |
| Other causes | 5682 | 28·19 |

The periods of life of the persons upon whom inquests were held, were: under 7 years, 5496—27·3 per cent.; above 7 years and under 16, 1716—8·5 per cent.; 16 years and under 60, 9731—48·3 per cent.; above 60 years, 3214—15·9 per cent.

Of the suicides, 960 were males and 389 females; and of those who died from excessive drinking, 229 were males and 94 females.

From 1848 to 1855, the variation in amount of commitments for trial was not great, and if this "be considered in relation to the undoubted constant increase of the aggregate of population and wealth," it "must be held to indicate a decreasing ratio of criminality." It is important to know that this "stationary character," as Mr. Redgrave terms the comparatively slight variations existing in the number of commitments during the period referred to, is also contemporaneous with a marked diminution in the severity of the punishments awarded to offenders. Since 1855 a very considerable decrease of commitments has taken place, in consequence of the operation of the Criminal Justice Act of 1855, which enlarged the summary jurisdiction of the magistrates. In 1857, an increase to the extent of 4·3 per cent. occurred upon the commitments of the previous year, and this increase extended over 32 counties; and as several of these had an effective police, the increase could not be attributed to the extension of the police force which had taken place in the kingdom during the year. This increase seems to have arisen in the great seats of manufacture and trade. It was most considerable in Lancashire (21·5 per cent.), Yorkshire (5·3 per cent.), and Cheshire (3·9 per cent.); very slight in Cumberland, Northumberland, and Durham; slight also in Leicestershire, Nottinghamshire, Warwickshire, and Worcestershire; but there was a slight decrease in Derbyshire, Staffordshire, and Gloucestershire. In the agricultural districts, the results were more mixed. There was an increase in Lincoln, Norfolk, Suffolk, Cambridge, Hereford, Berks, and Hants; a decrease in Essex,

Northampton, Bedford, Oxford (considerable), Bucks, Sussex, Wilts, Dorset, and Somerset. "In the metropolis, where any change affecting the working population is not so immediately felt, the decrease in Middlesex shown in the two previous years still continues, but not to the same extent; while in Surrey and Kent, a large portion of the population of which is located in and on the boundaries of the metropolis, the commitments increased." (p. xii.) In Wales there was an increase in seven counties; it was most marked in Glamorganshire. Of the border counties of the Province, an increase was shown in Monmouth and Hereford—large in the former county—and a considerable decrease in Shropshire.

The state of the different classes of crime, in reference to the slight increase of 1857, was as follows:—*Offences against the person* have been gradually increasing during the last three years, and in 1857 they increased 12·4 per cent., but entirely on the lesser offences—the common assaults. *Violent offences against property* have slightly increased since 1853, but the increase of 1857 was trifling. *Offences against property without violence* have decreased about 36 per cent. since the passing of the Criminal Justice Act, but there was an increase of 3·3 per cent. in 1857 upon the previous year. *Malicious offences against property* had increased, but in 1857 they were below the average of the last ten years. *In forgery and offences against the currency*, there was a decided increase in 1857; and *Miscellaneous offences* continued without much variation, "the chief noticeable fact being an increase last year (1857) of perjury, though the numbers are still much below the higher average which arose in 1851, when parties to suits were rendered liable to give evidence."

"Since 1848 there has not been a single commitment for any offence against the Crown or Government—nothing bearing the stamp of treason, sedition, or seditious riot. The year just past has been one of great trial to the labouring population; large numbers have been out of work, numbers 'on strike;' but it is proper to mention to their credit, and as an instance of their improvement, that there has been no recurrence of the seditious meetings or riotous disturbances which have heretofore almost uniformly attended such times of suffering and difficulty." (p. xiii.)

Of the persons committed, 4927 were, on trial, acquitted and discharged; 19 were acquitted on the ground of insanity; 16 were found insane (making a total number of 35 detained as insane); and 15,307 were convicted—of whom 2 in 1000 were sentenced to death; 0·7 per cent. to transportation, 16·2 per cent. to penal servitude, now the sole great secondary punishment; and 82·8 per cent. to imprisonment for various periods.

"The capital convictions," writes Mr. Redgrave,

"decreased last year to the average of the years immediately preceding 1856. They were for the following offences, to each of which the decrease extended, except to the robberies which under this head are confined to the violent class which alone continues capital:—

| OFFENCES. | 1857. | 1856. | 1855. | 1854. | 1853. | 1852. | 1851. | 1850. | 1849. | 1848. |
|---|---|---|---|---|---|---|---|---|---|---|
| Murder . . . . . . . | 20 | 31 | 11 | 11 | 17 | 16 | 16 | 11 | 19 | 23 |
| Attempts to murder, attended by dangerous bodily injuries . . . . . . | 9 | 10 | 10 | 7 | 8 | 11 | 6 | 8 | 12 | 6 |
| Sodomy . . . . . . | 10 | 11 | 20 | 15 | 9 | 14 | 18 | 14 | 18 | 18 |
| Burglary, with violence to persons . . . . . . . | 6 | 13 | 2 | 4 | 6 | 13 | 15 | 5 | 10 | 7 |
| Robbery, attended with wounds . . . . . . . | 7 | 2 | 5 | 7 | 14 | 5 | 13 | 10 | 3 | 3 |
| Arson of dwelling-houses, persons being therein . . | 2 | 2 | 2 | 5 | 1 | 2 | 2 | 1 | 4 | 3 |
| Total . . . | 54 | 69 | 50 | 49 | 55 | 61 | 70 | 49 | 66 | 60 |

"The executions last year (as in the previous sixteen years, with one exception,) were all for murder. Of the twenty persons convicted of this crime, thirteen were executed; their names follow, with such brief particulars of the circumstances of their crimes as could be accurately ascertained. I cannot avoid the remark, which arises in going through these cases, upon what slight and sudden provocations such heinous crimes appear in five instances to have been committed, while in four cases (including two of the preceding) the murderer was under the influence of drink, and in two instances had been actually drinking with his victim. In four cases the wife of the murderer was his victim.

"*Cheshire.*—John Blagg, aged 47. Murder of a gamekeeper by a poacher, supposed from revenge, not in an affray. *Essex.*—Michael Crawley, aged 62. Murder of his wife, arising out of a sudden quarrel. Charles Finch, aged 26. Murder of a young woman with whom he had cohabited; motive not very apparent. *Kent.*—George Kebble Edwards, aged 18. Murder of his brother, supposed in his sleep, from revenge for having reproached him with his idle, dissolute life. George Bave, aged 26. Murder by a seaman in her Majesty's service of a corporal of Marines, upon whose report he had been reduced. Stephen Fox, aged 23. Murder, from passionate revenge, of a young female to whom he was engaged, on her discarding him for his improper conduct with another woman. *Lancaster.*—Edward Hardman, aged 28. Murder of his wife, from a desire to marry another, and from religious differences. Henry Rogers, aged 37. Murder by the master of a merchant ship of one of his crew, by continued acts of barbarous treatment. *Middlesex.*—Robert Thomas Davis, aged 40. Murder of his wife in a sudden paroxysm of drunken jealousy. *Somerset.*—Thomas

Nation, aged 22. Murder of a companion with whom he had been drinking, to rob him of five pounds. John Beale, aged 30. Murder and robbery of a young woman with whom he had formerly lived as a fellow-servant; having enticed her from her situation under pretext of marriage, but with the design to plunder her of her reputed savings. *Stafford.*—George Jackson, aged 20. Murder and robbery committed under the influence of drink. *Glamorgan.*—John Lewis, aged 39. Murder of his wife; supposed in an attempt to obtain the possession of half a sovereign, to continue a drunken fit.

"To this list must be added the name of the following convict, who was convicted in December, 1856, but whose execution was respited (and did not take place till July, 1857): *Kent.*—Thomas Mansell, aged 28. Murder by a private soldier of his corporal, upon very groundless revenge, on small provocation." (pp. xv.—xvi.)

The prison returns show an increase of commitments to County and Borough prisons, and the Government prisons, in 1857, to the extent of 6·9 per cent. as compared with the previous year, and this notwithstanding the great falling-off in the commitments under the Mutiny Acts, consequent upon the termination of the war. The chief increase occurred in summary convictions, and in the commitments of debtors and on civil process. These latter commitments—which had decreased, under the operation of the Acts relating to proceedings for debt, to an average of 3621 for the three years 1845-7—have, since that date, continuously increased, until they reached 14,339 in 1857—25·7 per cent. above the preceding year; and they now form 10 per cent. of the whole number committed to prison. "This large and steady increase arises from the operation of the County Courts Acts, which, while they give no direct powers to arrest and imprison for debt, authorize the judges to commit for any period not exceeding forty days a defendant who refuses or neglects to pay a debt or damages on judgment obtained against him." (p. xxi.)

One test of the effects of prison discipline in the reformation of offenders is the number of previous commitments. The information upon this point is still defective. "It is, however, shown that 29·6 per cent. of the total committed last year (1857) had been previously in prison." The numbers recommitted as far as ten times were as follows:—

| Previous Commitments. | Proportion per cent. | Previous Commitments. | Proportion per cent. |
|---|---|---|---|
| Once | 13·0 | Five times | 1·3 |
| Twice | 5·7 | Seven times and above five | 1·6 |
| Thrice | 3·1 | Ten times and above seven | 1·2 |
| Four times | 2·1 | Above ten times | 1·7 |

The ages of the persons committed "mark strongly the large proportion of the young in the criminal class, though the temptation to the female arises at a rather more advanced period than the male." The proportion of each sex per cent. was:—

| | Under 21. | 21 to 30. | 30 and above. |
|---|---|---|---|
| Males . . . . | 35·9 | 30·6 | 33·5 |
| Females . . . . | 28·9 | 35·2 | 35·9 |

The proportion per cent. of prisoners at each period of life in the commitments was as follows:—

| Ages. | Proportion per cent. | Ages. | Proportion per cent. |
|---|---|---|---|
| Under 12 years . . . . . . | 1·5 | 40 years and under 50 . . . | 9·8 |
| 12 years and under 16 . . . | 8·5 | 50 years and under 60 . . . | 4·2 |
| 16 years and under 21 . . . | 24·0 | Above 60 years . . . . . . | 2·2 |
| 21 years and under 30 . . . | 31·8 | Age not ascertained . . . | ·3 |
| 30 years and under 40 . . . | 17·7 | | |

There was a marked decrease in the number of commitments under 16 years of age. This may in part be accounted for by the protracted detention of young criminals in Reformatories, who would otherwise swell the prison returns by their repeated commitments for short terms.

The country of birth was ascertained on the whole of the commitments within 1·4 per cent., and with the following results:—

| Birthplace. | Proportion per cent. | Birthplace. | Proportion per cent. |
|---|---|---|---|
| England . . . . . . . | 77·8 | Ireland . . . . . . . . | 14·5 |
| Wales . . . . . . . . | 2·3 | Colonies and East Indies . . | 0·5 |
| Scotland . . . . . . . | 1·9 | Foreign countries . . . . | 1·6 |

These results "are chiefly remarkable in the large proportion of prisoners of Irish extraction. They amount to 14·5 per cent. on the total commitments, and equal 1 in 362 of the population of Ireland; while the proportion from Scotland is only 1·9 per cent. on the total commitments, or 1 in 1204 of the population of that country." (p. xxiv.)

The returns of the degree of instruction among prisoners (excepting the debtors and military prisoners) show that 35·5 per cent. could neither read nor write; 58·0 per cent. could read, or read and write imperfectly; 5·1 per cent. could read and write well; 0·3 per cent. had a superior degree of instruction; and of 1·1 per cent. the degree of instruction was not ascertained. These returns differ only, in comparison with previous years, in showing a slight tendency to an increase of prisoners of the instructed class.

The occupations of those committed are shown in the following table; and it will be seen that the great majority of the commitments are from the uneducated and unskilled:—

| Occupations. | Males. | Females. | Total. | Proportion per cent. |
|---|---|---|---|---|
| No occupation . . . . . | 10,306 | 17,413 | 27,719 | 22·2 |
| Domestic servants . . . . | 1,252 | 3,504 | 4,756 | 3·8 |
| Labourers, charwomen, needle-women . . . . . . . . | 42,152 | 8,062 | 50,214 | 40·2 |
| Factory workers . . . . . | 4,588 | 2,033 | 6,621 | 5·3 |
| Mechanics and skilled workers | 22,332 | 659 | 22,991 | 18·4 |
| Foremen and overlookers of labour . . . . . . . . | 177 | 8 | 185 | 0·2 |
| Shopmen, shopwomen, clerks, &c. . . . . . . . . | 1,379 | 75 | 1,454 | 1·2 |
| Shopkeepers and dealers . . | 2,562 | 1,414 | 3,976 | 3·2 |
| Professional employments . . | 281 | 21 | 302 | 0.2 |
| Sailors, mariners, soldiers . . | 5,073 | — | 5,073 | 4·1 |
| Occupations not ascertained . | 975 | 557 | 1,532 | 1·2 |
| Total . . . . | 91,077 | 33,746 | 124,823 | 100·0 |

Mr. Redgrave has the following important remarks upon female committals:—

"For several years the increasing proportion of the female committals has been the subject of remark, and is a discouraging sign among some evidences of improvement which the returns present. Of the commitments for trial in 1857, the proportion of females was 21·0 per cent.; and of the summary convictions 28·3 per cent.; of the total commitments 24·3 per cent. But the females form a very much larger proportion of the re-commitments, and prove the greater difficulties in the way of female reformation after the taint of commitment to prison. It has just been stated, that of the commitments 24·3 per cent. are females, but of the re-commitments no less than 32·6 per cent. are females, and the case is aggravated as the number of re-commitments increases; for after the seventh previous commitment the number of females exceeds the males, and in the highest grade ascertained, ten times and above, is more than double the number, and yet it must be remembered, the female is little more than 1 to 4 of the total committed. With regard to age, it will be found that the career of crime does not begin so early in the female as the male. Under 16 years of age, the proportion of females to males is 13·4 per cent. only. In the five years between that age and 21 years, the proportion is doubled—26·9 per cent. But the largest proportion of females is found between the age of 21 and 30 years, when it reaches 29·9 per cent. In the whole of the remaining period of life, 30 years and above, the proportion falls to 28·3 per cent. In instruction the females are behind the males. 18·8 per cent. only of those who can 'read and write well' are females, while 30·7 per cent. could 'neither read nor write.' The chief fact with regard to country is, that while of the natives of England the females were 25·0 per cent., of the large number of natives of Ireland who appear in prison returns, the proportion of females was 38·5 per cent." (p. xxv.)

The rapid increase of the Reformatory system is evidenced by the increase of the numbers of children under detention from 594

to 1528 during the course of the year. Of those committed in 1857, numbering 1119, 429 could neither read nor write; 441 could read, or read and write imperfectly; 194 could read and write well; 7 had a superior degree of instruction; and of 48 the degree of instruction was not ascertained. 61 of the children were under 10 years of age, and none exceeded 16 years of age. The unprotected state of these children may be gathered from the following summary of their social condition on admission:—"Of 98, both parents, and of 309, one parent was dead; of 19, both parents, and of 37, one parent had deserted them; of 5, both parents, and of 32, one parent was in prison; and 130 were otherwise without the control of both parents, and 82 of one parent; so that of the 1119 committed during the year, 252 were entirely without parental care and control, and 460 had lost the care of one parent." (p. xxxvi.)

The criminal lunatics, the returns concerning which conclude the first part of the Blue-book, comprise—

"1. Those who are found by juries to have been insane at the time of committing the offences with which they were charged. 2. Those who on being arraigned for any offence were found to be then insane and unfit to be tried by a jury empanelled to determine that issue; or those who on their commitment for trial are, to avoid the necessity of this course, certified to the Secretary of State to be insane under stat. 3 and 4 Vict., c. 54. 3. Those committed by justices, who are apprehended under appearances of insanity, denoting a purpose to commit crime, and are without proper control. And 4. Those removed from prisons who have become insane during their confinement. All these lunatics are removed to lunatic asylums, hospitals, or licensed houses, under the orders made by the Secretary of State, and their detention under different forms is for their safe custody so long as they continue insane. The numbers so confined in the year ended the 29th September, 1856, were—

| | Males. | Females. | Total. |
|---|---|---|---|
| Under detention at commencement of the year . . . . . . . . | 457 | 129 | 586 |
| Received from other asylums . . | 26 | 6 | 32 |
| Committed in the year . . . . | 100 | 31 | 131 |
| Total . . . . . . | 583 | 166 | 749 |
| Discharged or removed; viz.: | | | |
| Died . . . . . . . . . | 33 | 6 | 39 |
| Escaped . . . . . . . . | 7 | — | 7 |
| On becoming sane . . . . | 12 | 7 | 19 |
| Removed, sane, for trial or punishment . . . . . . | 27 | 7 | 34 |
| Removed to other asylums . | 26 | 6 | 32 |
| | 105 | 26 | 131 |
| Remaining under detention . . . | 478 | 140 | 618" |

(p. xxvi.)

The offences with which these lunatics were originally charged are shown in the following table :—

| Offences. | Number of Persons. Males. | Females. | Total. | Offences. | Number of Persons. Males. | Females. | Total. |
|---|---|---|---|---|---|---|---|
| | | | | Forward | 416 | 131 | 547 |
| Murder . . . . | 85 | 40 | 125 | Fraud & embezzlement . . . . | 1 | — | 1 |
| Attempts to murder maim, &c. . . | 70 | 6 | 76 | Receiving . . . | 1 | — | 1 |
| Concealing birth, and infanticide . | — | 5 | 5 | Arson & malicious burning . . . | 28 | 4 | 32 |
| Manslaughter . . | 2 | 2 | 4 | Other malicious offences . . . . | 5 | 4 | 9 |
| Rape . . . . . | 9 | — | 9 | | | | |
| Unnatural offences | 11 | — | 11 | Forgery . . . . . | 3 | — | 3 |
| High Treason . . | 3 | — | 3 | Uttering counterfeit coin . . . | 3 | — | 3 |
| Seditious language | — | — | — | | | | |
| Assaults . . . | 25 | 5 | 30 | Breach of the peace | 19 | 5 | 24 |
| Indecent exposure of the person . . | 3 | — | 3 | Vagrancy . . . | 33 | 10 | 43 |
| Burglary & housebreaking . . | 30 | — | 30 | For want of sureties . . . . . | 29 | 6 | 35 |
| Robbery on the highway, &c. . . | 6 | 2 | 8 | Dangerous persons at large . . . | 1 | — | 1 |
| Sheep stealing . . | 9 | — | 9 | Insane wandering abroad without control . . . | 1 | — | 1 |
| Horse stealing . . | 4 | — | 4 | | | | |
| Cattle stealing . . | 1 | — | 1 | Deserters & under court-martial . . | 1 | — | 1 |
| Larceny — by servants, from person, &c. . . . | 158 | 71 | 229 | Other offences . . | 42 | 6 | 48 |
| | | | | Total . . . | 583 | 166 | 749 |
| | 416 | 131 | 547 | | | | |

Of these lunatics, 124 were found insane by jury; 15 were acquitted, insane; 55 were certified to be insane, or committed as such by justices; 4 were committed as dangerous lunatics by justices; and 551 were detained under order of the Secretary of State.

The detention of criminal lunatics in an asylum depending upon their return to sanity, the period of their confinement is necessarily undefined. Of the criminal lunatics under detention in 1857, 189 had been in confinement one year or under; 107, two years and above one; 66, three years and above two; 123, five years and above three; 141, ten years and above five; 62, fifteen years and above ten; 36, twenty years and above fifteen; and 25 above twenty years.

Of those lunatics who had been in confinement upwards of fifteen years, 18 had been guilty of murder, 15 of attempts to murder or maim, 1 of manslaughter, 1 of an unnatural offence, 3 of assaults, 1 of indecency, 1 of burglary, 14 of larceny, 4 of arson, 1 of uttering counterfeit coin, 1 of a breach of the peace, and 1 had been committed to prison for want of sureties.

The total cost of this class of prisoners in 1857 amounted to £19,836 9*s.* 6*d.*

---

### Art. VI.—ON CONVULSION.

